# GO2MSIG, an automated GO based multi-species gene set generator for gene set enrichment analysis

**DOI:** 10.1186/1471-2105-15-146

**Published:** 2014-05-17

**Authors:** Justin Andrew Christiaan Powell

**Affiliations:** 1 Takeda Cambridge Ltd, 418 Cambridge Science Park, Milton Road, Cambridge CB4 0PZ, UK

**Keywords:** Gene set enrichment analysis (GSEA), GO ontology, Gene set collection, ErmineJ

## Abstract

**Background:**

Despite the widespread use of high throughput expression platforms and the availability of a desktop implementation of Gene Set Enrichment Analysis (GSEA) that enables non-experts to perform gene set based analyses, the availability of the necessary precompiled gene sets is rare for species other than human.

**Results:**

A software tool (GO2MSIG) was implemented that combines data from various publicly available sources and uses the Gene Ontology (GO) project term relationships to produce GSEA compatible hierarchical GO based gene sets for all species for which association data is available. Annotation sources include the GO association database (which contains data for over 200000 species), the Entrez gene2go table, and various manufacturers’ array annotation files. This enables the creation of gene sets from the most up-to-date annotation data available. Additional features include the ability to restrict by evidence code, to remap gene descriptors, to filter by set size and to speed up repeat queries by caching the GO term hierarchy. Synonymous GO terms are remapped to the version preferred by the GO ontology supplied. The tool can be used in standalone form, or via a web interface. Prebuilt gene set collections constructed from the September 2013 GO release are also available for common species including human. In contrast human GO based sets available from the Broad Institute itself date from 2008.

**Conclusions:**

GO2MSIG enables the bioinformatician and non-bioinformatician alike to generate gene sets required for GSEA analysis for almost any organism for which GO term association data exists. The output gene sets may be used directly within GSEA and do not require knowledge of programming languages such as Perl, R or Python. The output sets can also be used with other analysis software such as ErmineJ that accept gene sets in the same format. Source code can be downloaded and installed locally from http://www.bioinformatics.org/go2msig/releases/ or used via the web interface at http://www.go2msig.org/cgi-bin/go2msig.cgi.

## Background

High throughput expression profiling using either array or sequencing based methods often generates noisy data. Reducing the noise levels by increasing the number of replicates can be precluded by cost considerations. Clear biological signals may be present in such data that are not readily visible when taking a gene by gene approach to the analysis. An example would be one where all 10 genes in a known bio-synthetic pathway have become up-regulated, each by a small amount that would be seen as experimental noise when considered in isolation, but may be significant given prior knowledge that the pathway exists. Consequently recent years have seen the rise of gene set based methods which take pre-defined gene sets and look for coordinated gene expression changes using various statistical methods [1-6]. As well as increasing sensitivity, gene set based approaches also read out directly in terms of ‘systems’ - one step further towards the overview that the researcher would anyway be attempting to synthesise from the individual fold changes.

Gene sets can be generated in a number of ways ranging from the highly manual to the automated. Due to the size of a typical gene complement, manual generation of a comprehensive collection of gene sets is time consuming.

One example of an automated approach to gene set generation is to use the associations between particular Gene Ontology (GO) project [7] terms and the genes of the organism in question. Such association data is available from a variety of sources, either automatically or manually curated. A gene set can be constructed for each GO term, with members comprising those genes annotated with that term and potentially, as discussed later, with child terms of that term. Thus the Gene Ontology supplies the gene set functional definitions, and the association data source supplies the gene membership of those sets. GO terms have biological meaning, and the co-ordinated perturbation of a set of genes whose common attribute is annotation with the same term (be it related to a pathway, cellular location, or biological system) is clearly informative.

One widely used gene set based analysis technique is Gene Set Enrichment Analysis (GSEA) [8,9]. GSEA has been implemented in a number of forms including, importantly, a freely available standalone desktop-based implementation written in Java and available from the Broad Institute that enables gene set based analysis to be carried out by non-experts. This implementation is a sophisticated platform, with many tuneable parameters, comprehensive statistical output and additional tools such as ‘leading edge analysis’ for interrogation of the results. Unlike many existing GO-specific enrichment packages GSEA does not require an arbitrary significance cut-off to be made, and also permits the use of sample permutation rather than gene permutation to assess the significance of the identified perturbed gene sets. These two features allow it to be more sensitive and less susceptible to false positives than simpler enrichment algorithms [10]. A GSEA analysis requires the experimental data set, a predefined gene set collection, and potentially a mapping between the identifiers used. A number of pre-defined gene set collections formatted for the Broad Institute GSEA implementation are available from the Molecular Signatures Database (MSigDB) [9], including the ‘c5’ GO based gene set collection for human that dates from September 2008. However, updated human GO based gene sets or GO based gene sets for other species (and indeed significant numbers of *any* gene sets for other species) are not available from MSigDB and have to be constructed *ad-hoc*. Here I present GO2MSIG, a tool and database implementation which uses data from the GO consortium, NCBI or array manufacturer annotation files to enable the researcher to generate MSigDB compatible gene set collections for many species without the need for custom software writing or manual gene set curation. The tool takes various parameters for fine tuning the output sets, and is available in standalone form and via the web. The resultant gene set collections may be used directly with the Broad Institute GSEA implementation, or with other tools such as ErmineJ [3] that also accept MSigDB format gene set collections.

## Implementation

A GO project ontology is represented by an directed acyclic graph (DAG), each term being a node in the graph. Each parent term can have multiple children, and each child term can have multiple parents. There is a single root term for each of the three ontologies, ‘molecular function’, ‘biological process’ and ‘cellular component’. Terms become more specific as one moves away from the root.

Often only the more specific GO terms are annotated in the association databases. For instance in the September 2013 release of the Entrez Gene database gene2go table [11] only 16 mouse genes are directly annotated with the cellular component term ‘sarcomere’. However a total of 81 additional genes are annotated with child terms of ‘sarcomere’ such as ‘A band’, ‘I band’ or ‘Z disc’. Absence of the higher level annotations means that in this case the ‘sarcomere’ gene set will be missing the vast majority of sarcomere related genes and the sensitivity of GSEA towards perturbations in sarcomere biology will be significantly reduced. Making a fully featured gene set collection in which these implicit but highly meaningful associations are captured requires that the GO DAG is used to annotate genes with these inferred higher level term associations. Thus both the association data and the rich structure of the ontology contribute to the extraction of meaning from the data. Gene annotation with these inferred associations is equivalent to propagation of the explicit term associations up the DAG towards the root. The original Broad Institute ‘c5’ collection was constructed in this way (A. Liberzon, personal communication). Other GO based gene set tools adopting a similar strategy include FatiGO [1], ErmineJ [3], GoParGenPy [12], GoStat [13] and DAVID [14]. Likewise GO2MSIG includes a propagation phase during set construction.

**Figure 1:**
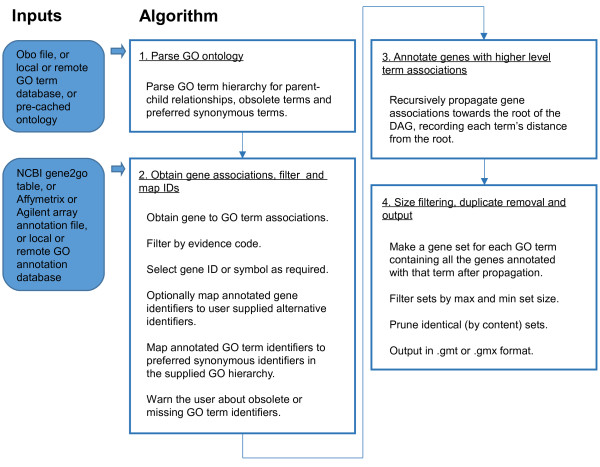
**Work flow of GO2MSIG.** Input data is shown in filled blue boxes, algorithmic steps are shown in unfilled boxes.

Figure 1 shows in detail the procedure adopted by GO2MSIG. The program flow can be broken down into 4 main phases. The first is obtaining the GO term hierarchy, the second is obtaining the gene associations, the third is the propagation of the associations towards the root term and the fourth is post-processing the resultant gene sets and formatting the output. Each phase consists of a number of discrete steps, shown in Figure 1 and described below in order of execution.

### Obtaining and parsing the GO term hierarchy

GO2MSIG can obtain term information (the IDs, names and hierarchical relationships of the GO terms) from a standard GO term database (installed locally or remotely) or an OBO flat file. If used with a slow implementation of the GO term database GO2MSIG can cache the GO hierarchy in order to speed up future calls. The GO ontology is constantly evolving, hence the GO database contains information about GO terms now obsolete, and about terms which are synonymous with other terms that are now preferred. GO2MSIG parses the database to determine the list of current terms and their parent–child relationships, the list of obsolete terms and the list of terms with synonymous preferred terms.

### Obtaining, parsing, filtering and mapping the GO term gene associations

GO2MSIG can obtain GO term gene associations from any of three sources: Affymetrix or Agilent array annotation files, the GO association database, or the NCBI curated Entrez Gene database gene2go table [11]. The Entrez Gene database has the advantage over the GO association database that it uses a consistent gene identifier (the Entrez Gene ID), but the disadvantage that it contains fewer gene associations. The Entrez Gene database is the source of the pre-built MSigDB GO sets for human.

The next step allows the user to filter the associations by evidence code. The association between GO terms and genes is accompanied by an evidence code which describes how strong the evidence is for that association. IEA (inferred from electronic annotation) for example means that the association has been inferred automatically, whereas TAS (traceable author statement) means that the association has been proven experimentally and that the assertion can be located in a paper. The original MSigDB human GO sets used a subset of evidence codes ensuring only well supported associations were used. The same defaults are used by GO2MSIG, but any combination can be specified by the user. For less well characterised species it can be that only IEA supported associations exist.

Following evidence filtering, GO2MSIG selects either the gene symbol or the Entrez Gene ID as specified by the user (for those association data sources where this is an option).

The next step allows the user to supply an optional file remapping the gene identifiers obtained from the association data source to user supplied identifiers. This may be necessary if the association data uses a different type of gene identifier to those present in the experimental results. One common use would be to map from probe IDs supplied in an array annotation file to gene IDs. The program can be set to either leave identifiers missing from the translation file unmapped, or to repress them. The former is useful if the user wishes to remap only a subset of gene identifiers, useful in those cases where the annotation data source is inconsistent in its identifier use. The latter can be useful when trying to extract gene sets for a single species from an Affymetrix annotation spreadsheet that contains more than one species.

Finally GO2MSIG remaps synonymous GO terms to the term preferred by the version of the GO hierarchy being used. Obsolete terms in the associations are ignored. Informational warnings are issued when synonymous or obsolete terms are encountered, and also when GO terms in the gene association data source are not represented in the GO term data source at all (possible if the term data source is older than the association data source).

### Annotation of genes with higher level term associations

The annotation of genes with higher level term associations is performed by recursively propagating gene associations explicitly defined in the association database up the DAG towards the root. During this process the shortest path connecting each term to the root is recorded for later output.

### Size filtering, duplicate removal and output

The first step after propagation is filtering the collection of sets by user selectable maximum and minimum size cut-offs. The minimum cut-off prevents the output of many very small gene sets that would never achieve statistical significance. The maximum cut-off removes GO terms that are so general that the results would be meaningless.

Gene set collections built from GO will likely contain multiple GO terms with identical gene associations. Since such duplicate gene sets can affect the accuracy of the key GSEA false discovery rate statistic, the next step is to collapse these down to a single set. The original MSigDB gene set collections eliminate identical terms leaving one representative. Elimination of identical terms without record can make inference harder. If, for example, a parent term ‘muscle system process’ has the same gene associations as its eliminated child term ‘muscle contraction’, then during analysis the user looking at the GO hierarchy will likely want to know whether the significant change identified for ‘muscle system process’ is a result of ‘muscle contraction’, or other child terms such as ‘muscle hypertrophy’. This is hard to do if the fact that the child term existed and is identical to the parent term is not recorded. GO2MSIG also records each unique gene set (by gene content) only once. However the description field in the output file will contain a list of *all* GO terms with that identical set of gene associations. The URL link field (which can only reference one term) contains a link to whichever of the GO terms has the shortest distance between it and the root term - in other words the most general of the terms associated with that gene set.

During analysis of the results from a GO based gene set analysis the experimenter is likely to want to home in on the more specific terms that show statistically significant changes. In this implementation a rough guide to specificity is provided by appending the distance of each term from the root (calculated during the propagation stage) to the end of the term description during the final output. This is analogous to the concept of ‘levels’ used by the functional enrichment analysis application FatiGO [1].

Finally the set is output in either .gmt or .gmx format, one being essentially the transpose of the other.

## Results and discussion

Interrogating the GO and Entrez Gene databases with GO2MSIG itself revealed that these databases contain GO annotation data for over 200000 Eukaryotic and Prokaryotic species.

**Figure 2:**
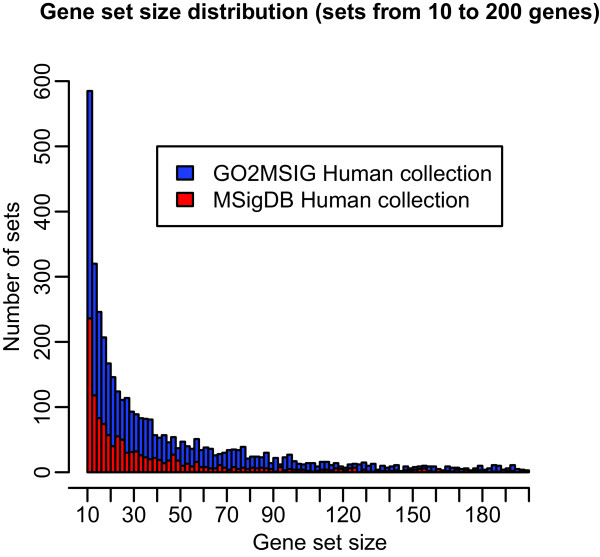
**Gene set size distribution.** Red bars show the distribution of gene set sizes in the MSigDB c5 collection for sets from 10 to 200 genes in size. Blue bars show the distribution for the equivalent collection generated by GO2MSIG. The 10 to 200 gene size range includes over 89% of the gene sets in each collection.

Although updated gene set collections were released by MSigDB in May 2013, the ‘c5’ GO based human gene sets are still derived from GO annotations dating from 2008. Figure 2 compares the MSigDB c5 collection by gene set size and number (filtered for sets containing between 10 and 200 genes) to the equivalent collection generated by GO2MSIG using annotation data dating from September 2013. The full GO2MSIG built collection (filtered to omit sets with fewer than 10 or more than 700 genes) contains 4403 gene sets with an average size of 81 genes (Table 1). In contrast the ‘c5’ set contains 1422 gene sets sized between 10 and 700 genes with an average size of 69 genes. Thus we see that building the collection from current data increases the collection size by more than a factor of 3.

**Table 1:** **Sizes of gene set collections built from the NCBI gene2go table**^**1**^

			**Number of gene sets in collection (average number of genes in set)**	
**Taxon ID**	**Organism**	**Number of genes with GO annotation**	**All evidence codes**	**High quality evidence codes**
234826	*Anaplasma marginale* str. St. Maries	196	48 (40)	
212042	*Anaplasma phagocytophilum* str. HZ	1288	218 (55)	221 (60)
3702	*Arabidopsis thaliana*	27942	2032 (129)	1951 (85)
227321	*Aspergillus nidulans* FGSC A4	7326	1152 (69)	35 (31)
198094	*Bacillus anthracis* str. Ames	5097	465 (81)	466 (81)
9913	*Bos taurus*	5567	2634 (67)	1285 (58)
6239	*Caenorhabditis elegans*	12642	1505 (84)	1098 (81)
195099	*Campylobacter jejuni* RM1221	1826	315 (62)	316 (63)
246194	*Carboxydothermus hydrogenoformans* Z-2901	2609	363 (64)	362 (65)
227377	*Coxiella burnetii* RSA 493	1798	271 (67)	272 (67)
214684	*Cryptococcus neoformans* var. neoformans JEC21	3427	969 (68)	
7955	*Danio rerio*	16957	2201 (83)	1342 (68)
243164	*Dehalococcoides ethenogenes* 195	1583	265 (72)	265 (71)
352472	*Dictyostelium discoideum* AX4	7694	1184 (86)	801 (72)
7227	*Drosophila melanogaster*	12560	2750 (83)	2459 (78)
205920	*Ehrlichia chaffeensis* str. Arkansas	1090	221 (56)	223 (59)
511145	*Escherichia coli* str. K-12 substr. MG1655	2518	198 (112)	
9031	*Gallus gallus*	2104	1460 (64)	643 (52)
243231	*Geobacter sulfurreducens* PCA	3269	347 (82)	348 (82)
9606	*Homo sapiens*	18106	5808 (82)	4403 (81)
265669	*Listeria monocytogenes* serotype 4b str. F2365	2811	384 (79)	385 (79)
243233	*Methylococcus capsulatus* str. Bath	2902	377 (72)	378 (72)
10090	*Mus musculus*	24667	5615 (79)	3643 (74)
222891	*Neorickettsia sennetsu* str. Miyayama	928	204 (54)	206 (56)
39947	*Oryza sativa* Japonica Group	4266	30 (18)	2 (14)
36329	*Plasmodium falciparum* 3D7	1770	212 (65)	219 (67)
223283	*Pseudomonas syringae* pv. tomato str. DC3000	3950	436 (73)	439 (77)
10116	*Rattus norvegicus*	18599	5746 (79)	3081 (75)
246200	*Ruegeria pomeroyi* DSS-3	4250	497 (85)	496 (86)
559292	*Saccharomyces cerevisiae* S288c	6244	2005 (75)	1849 (74)
284812	*Schizosaccharomyces pombe* 972 h-	5276	1627 (82)	1118 (67)
211586	*Shewanella oneidensis* MR-1	4272	418 (79)	419 (79)
999953	*Trypanosoma brucei brucei* strain 927/4 GUTat10.1	1073	157 (74)	147 (80)
9606	*Homo sapiens* (MSigDB collection)	18106		1422 (69)^2^
9606	*Homo sapiens* (From Affymetrix annotation file)	18106	5383 (80)	

Gene sets were built from the NCBI gene2go annotation table and GO ontology downloaded on 13^th^ September 2013. Default settings were used which filter out gene sets containing fewer than 10 or more than 700 genes. Organisms were omitted when the biggest collection contained fewer than 30 sets. In cases where use of all evidence codes *reduces* the number of gene sets compared with using high quality codes only, this is due to maximum set size filtering. ^1^For comparison the currently available MSigDB GO based human collection and a human set built from the annotation file for the Affymetrix HG-U133 Plus 2.0 array are also shown. ^2^Set number and sizes were calculated for the MSigDB collection with filtering as above (the full collection contains 1454 gene sets).

Tracing the origin of the gene associations in the GO2MSIG built human collection of 4403 sets showed that 83% of the gene to GO term associations are the result of propagation of gene annotations up the GO term hierarchy, rather than arising directly from the annotation databases. This shows that the propagation of gene annotations is essential to the production of properly comprehensive gene sets.

Non-human collections are not available from MSigDB, and are frequently not available elsewhere preformatted for direct use with Broad Institute GSEA. To illustrate the utility of GO2MSIG for other species, gene set collections were built for all organisms annotated in the NCBI gene2go table. The individual gene set collections could be computed by GO2MSIG in under 30 seconds on a standard laptop. Table 1 lists the number of gene sets in each collection and the average number of genes per set, for each organism. It also lists the number of genes annotated with one or more GO terms as a quick guide to the comprehensiveness of the available GO annotations for each organism. Using the GO project annotation database, array annotation files or other appropriately formatted annotation sources, such collections can be rapidly generated for many thousands of other species.

As a further comparison a human gene set collection was built from annotation data contained in the Affymetrix annotation file for the HG-U133 Plus 2.0 3′ expression array. A map file derived from the same annotation file was used to map probe IDs to gene symbols. The resultant collection contained 5838 gene sets. As the array annotation file does not contain evidence codes this is compared in Table 1 with the ‘all evidence code’ sets. In this case 75% of the gene associations in the collection resulted from propagation of associations up the GO term hierarchy. Release 33 of the annotation file was used which dates from October 2012, whereas the GO term database dates from September 2013. During the build 37 GO terms from the annotation file were automatically replaced with a more up-to-date synonym and 60 GO terms were discarded due to obsolescence. Thus array annotation files can be used in the same way as the NCBI or GO databases to build comprehensive gene set collections.

## Conclusions

This paper describes an easy-to-use program which enables rapid generation of GSEA compatible gene set collections from a variety of data sources and for many organisms. Using GSEA with these collections can rapidly uncover biologically meaningful patterns in array or sequence based gene expression data sets from species for which such analyses previously would have been significantly more time consuming. The easiest way to make use of this program is via the GO2MSIG website, which obviates the need to install MySQL databases or make calls to potentially slow external GO database mirrors. All gene2go derived gene sets shown in Table 1 are available for download from http://www.go2msig.org/cgi-bin/prebuilt.cgi.

## Availability and requirements

**Project name:** GO2MSIG

**Project home page:**http://www.bioinformatics.org/go2msig/

**Operating system:** Platform independent

**Programming language:** PERL

**Other requirements:** GO::Parser library. MySQL if using local databases.

**License:** GNU GPL v2

**Any restrictions to use by non-academics:** None

## Competing interests

The author declares that he has no competing interests.

## Authors’ contribution

JP designed, implemented and tested the algorithm as a Perl script, wrote the documentation, set up the website and wrote the manuscript.
